# What Can Psychiatric Disorders Tell Us about Neural Processing of the Self?

**DOI:** 10.3389/fnhum.2013.00485

**Published:** 2013-08-15

**Authors:** Weihua Zhao, Lizhu Luo, Qin Li, Keith M. Kendrick

**Affiliations:** ^1^Key Laboratory for Neuroinformation, School of Life Science and Technology, University of Electronic Science and Technology of China, Chengdu, China

**Keywords:** schizophrenia, autism spectrum disorder, major depression, borderline personality disorder, self-processing, cortical midline system, mirror neuron system, inter-hemispheric connectivity

## Abstract

Many psychiatric disorders are associated with abnormal self-processing. While these disorders also have a wide-range of complex, and often heterogeneous sets of symptoms involving different cognitive, emotional, and motor domains, an impaired sense of self can contribute to many of these. Research investigating self-processing in healthy subjects has facilitated identification of changes in specific neural circuits which may cause altered self-processing in psychiatric disorders. While there is evidence for altered self-processing in many psychiatric disorders, here we will focus on four of the most studied ones, schizophrenia, autism spectrum disorder (ASD), major depression, and borderline personality disorder (BPD). We review evidence for dysfunction in two different neural systems implicated in self-processing, namely the cortical midline system (CMS) and the mirror neuron system (MNS), as well as contributions from altered inter-hemispheric connectivity (IHC). We conclude that while abnormalities in frontal-parietal activity and/or connectivity in the CMS are common to all four disorders there is more disruption of integration between frontal and parietal regions resulting in a shift toward parietal control in schizophrenia and ASD which may contribute to the greater severity and delusional aspects of their symptoms. Abnormalities in the MNS and in IHC are also particularly evident in schizophrenia and ASD and may lead to disturbances in sense of agency and the physical self in these two disorders. A better future understanding of how changes in the neural systems sub-serving self-processing contribute to different aspects of symptom abnormality in psychiatric disorders will require that more studies carry out detailed individual assessments of altered self-processing in conjunction with measurements of neural functioning.

## Introduction

Brain mechanisms controlling our fundamental sense of self are not only of great interest in themselves but also fundamentally influence our social, cognitive, and emotional behaviors. For most of us that feeling of a secure, private sense of self that only we can access, and which affords the simple ability of distinguishing between ourselves and others, is a given. We use our sense of self to monitor, gage, and learn about our reactions to internal/external changes to our bodies as well as the actions we perform and their consequences on our physical and social environment. Thus any human mental disorder where fundamental aspects of self-processing are impaired is likely to also manifest associated impairments in social and emotional behavior.

There are many different definitions of the self and detailing them is beyond the scope of the present paper (see Kircher and David, 2003; Gillihan and Farah, 2005; Legrand and Ruby, 2009; Christoff et al., 2011). The most pragmatic approach is to distinguish the physical, or bodily self (i.e., knowledge and awareness of the body) from the mental, or psychological self (i.e., autobiographical knowledge, knowledge about personal traits, and experience of first-person perspective). Some consider the integration of these two aspects results in the sense of self as an agent (Gillihan and Farah, 2005), whereas others propose a broader definition which includes an individual’s knowledge of their relationship with physical and social stimuli in their environment (Northoff et al., 2006). Many studies investigating the neural circuitry involved in self-processing have used behavioral paradigms which focus on self attributes (i.e., “me”) as opposed to the self as an agent (“I”). It has been argued that both attribute and agency aspects of self-processing must be taken into account (Christoff et al., 2011) and could involve different neural systems. Additionally, cultural and learning influences on self-processing have been increasingly recognized with differences between more collectivist (Asian) and independent (Western) cultures having been highlighted, with the former having a more extended self-representation including other significant individuals, notably mothers (Zhu et al., 2007). Interestingly, this cultural effect is weakened in Chinese people raised in Western countries (Ng et al., 2010) and illustrates that both culture and learning can, to some extent, produce an extended concept and representation of self which can potentially blur the distinction between self and other processing.

**Figure 1:**
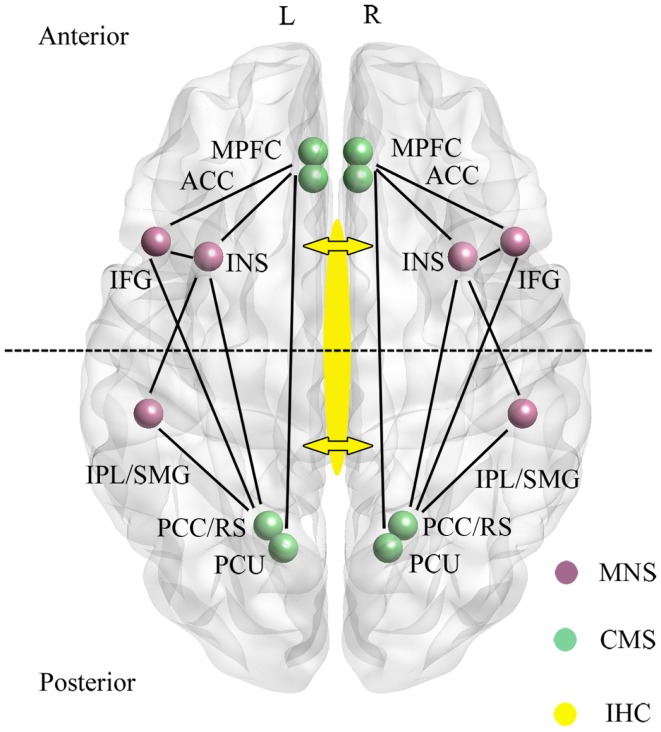
**Main regions and functional connections of the cortical midline (CMS) and mirror neuron (MNS) systems implicated in the control of self-processing**. Inter-hemispheric connectivity (IHC) via the anterior and middle/posterior corpus callosum are indicated by arrows with the dotted line across the two hemispheres sub-dividing the brain into anterior frontal regions interconnected via the anterior corpus callosum and posterior parietal regions interconnected via the middle/posterior corpus callosum. ACC, anterior cingulate; IFG, inferior frontal gyrus; INS, insula; IPL, inferior parietal lobule; mPFC, medial prefrontal cortex; PCC, posterior cingulate cortex; PCU, precuneus; RS, retrosplenial cortex; SMG, supramarginal gyrus. For simplicity the anterior (mPFC and ACC) and posterior (PCC/RS and PCU) are displayed as single regions. L, left and R, right hemisphere.

In recent years a large number of studies mainly using both resting-state and task-related functional magnetic resonance imaging (fMRI) studies in healthy human subjects have implicated a cortical midline system (CMS) involving frontal and parietal components of the default mode network (DMN) as being of key importance for “self” as opposed to “other” processing (see Qin and Northoff, 2011) (see Figure 1). The particular involvement of the DMN has received conceptual support due to the observation that regions within it are most active when subjects are at rest and their thoughts are internally directed due to an absence of external stimuli (Gusnard et al., 2001). As such the DMN is referred to as “task-negative” to distinguish it from anti-correlated “task-positive” networks which show low levels of activity at rest which then increase during the performance of tasks requiring attention to external stimuli (Fox et al., 2005). However, although many studies have provided evidence for regions in the DMN responding more to self than other-related stimuli, it has been difficult to disentangle the extent to which responses are truly self-specific or are influenced by other factors such as familiarity and learning.

A recent meta-analysis has identified CMS and other regions associated specifically with self-related information as opposed to familiar or unfamiliar others (Qin and Northoff, 2011). Evidence for self-specificity was mainly found in the perigenual anterior cingulate (AC), inferior frontal gyrus (IFG), and insula, with the AC localization corresponding to that shown in resting-state studies to be deactivated during tasks. The medial prefrontal cortex (mPFC) was influenced by both self and familiarity and the posterior cingulate cortex (PCC) by familiarity more than self. Thus overall, self-specific processing was associated more with frontal than parietal regions in the CMS.

A second neural system associated with aspects of self-processing, and which also includes fronto-parietal [IFG, precentral gyrus, precuneus, supramarginal gyrus (SMG), inferior parietal lobule (IPL)] as well as limbic (anterior insula and anterior mesial frontal cortex) regions is the so-called “Mirror Neuron System” (MNS) (Uddin et al., 2007; Cattaneo and Rizzolatti, 2009) (see Figure 1). The MNS responds equivalently to specific goal-directed actions whether they are performed by self or others, and is therefore ideally suited to compute self-other discriminations. Indeed, in parts of the MNS even auditory information suggesting that a specific action has been performed out of sight is an effective stimulus (Cattaneo and Rizzolatti, 2009). The CMS and MNS are linked both in frontal and parietal regions (particularly via the insula and IFG, although also between the IPL and PCC/precuneus), and it has been proposed that they have an integrated role whereby the MNS performs physical other-to-self mapping, important for understanding agency attribution, whereas the CMS is more important for understanding psychological aspects. Interestingly, the meta-analysis on CMS and DMN in relation to self carried out by Qin and Northoff (2011) also identified a degree of self-specific processing in the two MNS regions (IFG and insula) with the most extensive connections with the CMS. Indeed, a number of studies have shown that the insula is activated during self-reflection (Modinos et al., 2009).

There is considerable interest in establishing whether self-recognition and sense of agency occur independently in both brain hemispheres, or are lateralized to some extent, or dependent upon connectivity between the hemispheres. The anterior regions of the CMS (AC, mPFC) and MNS (IFG and insula) are connected via the anterior corpus callosum, whereas the posterior ones are connected via the medial/posterior callosum (PCC, precuneus, IPL, SMG) (see Figure 1). Studies using split-brain patients have generally concluded that either auditory or visual self-recognition can occur to some extent independently in both hemispheres, with some studies reporting a right hemisphere advantage and others left (see Uddin, 2011). On the other hand evidence from patients with “alien hand syndrome” (AHS – where individuals deny ownership of a hand and sometimes also its actions) suggests that some aspects of this syndrome are produced by damage either to the anterior (anarchic hand – where subjects consider that goal-directed movements of the hand are not under the control of their own will) or middle/posterior (inter-manual conflict) corpus callosum regions. While damage to frontal and parietal cortical regions may also contribute to aspects of AHS, there does seem to be support for the view that inter-hemispheric connectivity (IHC) involving the corpus callosum is important for awareness of goal-directed movement and a sense of limb ownership (see Uddin, 2011). Findings from patients with callosal agenesis also suggest impairments in aspects of sense of agency with poor personal insight, introspection, perspective taking, and self-awareness (Brown and Paul, 2000; Paul et al., 2007). Thus IHC may also play an important role in self-processing.

While we may be able to learn more about the neural substrates of self-processing from patients with brain lesions, they are relatively rare and damage often involves a number of different systems. A recent case study, for example, reported preserved self-awareness in a patient with damage to the insula, AC, and mPFC but without damage to the parietal lobes (Philippi et al., 2012). This might perhaps suggest that self-awareness does indeed involve multiple brain systems. However, a far more extensive source of patients with altered aspects of self-processing are those with psychiatric disorders. While these disorders are clearly complex, and often include extensive cognitive and emotional processing dysfunction, they are commonly associated with different patterns of altered functioning of CMS and MNS systems and IHC which may contribute to abnormal self-processing. Here we will focus on some of the main disorders where self-processing is known to be affected, namely schizophrenia, autism spectrum disorder (ASD), unipolar depression, and borderline personality disorder (BPD). To identify papers specifically addressing self-processing alterations in these disorders we used main search terms of “schizophrenia and self,” “autism and self,” “depression and self,” and “BPD and self” in PubMed and Google Scholar. Additionally we used search terms of “schizophrenia and sense of agency,” “depression and rumination,” and references included in the recent reviews cited.

## Self-Processing in Schizophrenia

Schizophrenia has long been considered as a self-disorder. However, although self-experience anomalies are considered to be a first-rank core symptom of schizophrenia (Schneider, 1959), there is still considerable debate as to their precise definition and the extent to which they contribute to other extensive cognitive and emotional dysfunction in the disorder as well as other symptoms such as pain insensitivity. Aspects of both the physical and psychological self are disturbed in schizophrenia with the main features being in terms of impaired self-other discrimination, including body-ownership, and also an altered sense of agency whereby patients have problems in determining whether their thoughts and actions are controlled by themselves or by external agents. It is not the purpose of this review to discuss the various symptoms in detail since this is done elsewhere (Sass and Parnas, 2003; Lysaker and Lysaker, 2010; Waters and Badcock, 2010). One of the key recent observations however is that evidence for self-disorders has been found in both schizotypal personality disorder and schizophrenia, suggesting that non-psychotic anomalies of self-experience occur across the schizophrenia spectrum (Raballo et al., 2011) and are a core feature which may also influence other cognitive and emotional symptoms. An influential theory attempting to explain the root cause of self-disorders in schizophrenia is that self-other distinctions are due to a faulty action processing mechanism linking motor and sensory systems (Frith et al., 2000; Waters and Badcock, 2010). This so-called internal forward model predicts the sensory consequences of motor commands which allows differentiation of sensations experienced on the basis of whether they result from intentional movement or from changes in the external world. Distinction between sensations is achieved because the model predicts reduced sensory effects occurring following self-generated movements and the internal system therefore deduces that sensations result from a self-generated motor command. Thus it is hypothesized that schizophrenia patients are impaired in their ability to discriminate between sensations resulting from their own self-generated motor actions and those resulting from external agents due to prediction errors occurring in this sensorimotor comparator system. The result of this is that patients can feel that their own actions are not generated by themselves but by some external agent. This has been shown experimentally using action perception/feedback tasks where a reduction in precision in predicting the sensory consequences of action is associated with the severity of delusions of control (Synofzik et al., 2010). This lack of precision results in patients placing greater reliance on external retrospective rather than internally generated predictive cues for linking actions with external events (Voss et al., 2010). A simple behavioral demonstration of this is self-tickling where healthy subjects know the action is self-generated and the sensory feedback completely predictable and so are unresponsive, whereas when we are tickled by someone else it is not so predictable and we do respond. However, in schizophrenia patients this self vs. other recognition distinction breaks down and they can effectively tickle themselves (Blakemore et al., 2000). A similar argument based on a deficit in a neural comparator linking perception and action impairment has been made to extend this concept into cognitive systems to explain impaired theory of mind and emotional response deficits (Frith et al., 2000; Jeannerod, 2003). Indeed, a recent study has reported evidence for a deficit in error-likelihood prediction in the mPFC in schizophrenia patients in the context of a working-memory task (Krawitz et al., 2011). Additional behavioral evidence for breakdown of a comparator mechanism linking perception and action during self-other interactions is impaired gesture and movement imitation (Matthews et al., 2013) and emotional contagion (unconscious imitation of smiling and yawning – Haker and Rössler, 2009) in patients. These behaviors are thought to particularly involve the MNS and therefore suggest that its function is altered in some way in schizophrenia.

A large number of structural and resting-state MRI studies have endeavored to establish the key structural and functional changes which occur in schizophrenia. Overall these studies consistently show that there is both structural and functional evidence for widespread disconnection between brain regions both within and across hemispheres and that changes in the CMS and MNS (Friston and Frith, 1995; Garrity et al., 2007; Huang et al., 2010; Lynall et al., 2010; Guo et al., 2012) and key sensorimotor and cortical integration regions such as the thalamus (Clinton and Meador-Woodruff, 2004; Welsh et al., 2010; Marenco et al., 2013) occur frequently as well as reduced IHC involving both the corpus callosum and the anterior commissure (Crow, 1998; Choi et al., 2011; Guo et al., 2012, 2013). Altered functional connectivity between pre-motor and motor cortices has also been observed (Guo et al., 2012), and may contribute to a disturbed sense of bodily self in schizophrenia which is known to be associated with these motor connections as well as the insula (Ferri et al., 2012a,b).

While there has been increasing recent interest in investigating which structural and resting-state changes in neural systems may specifically relate to the core symptoms of self-disorder in schizophrenia, many of the routine measures of positive and negative symptoms used do not address self-processing impairments adequately. For example, the widely used Positive and Negative Symptom Scale (PANSS) only considers the presence of hallucinations and delusions and lack of insight (lack of awareness of illness and need for treatment). The presence of auditory hallucinations is associated with more impaired self-recognition performance (Waters et al., 2012) and degree of preserved insight is associated with alterations in responses in brain regions associated with self-reflection (van der Meer et al., 2012). However, going forward the development and use of better qualitative and quantitative measures of self-disorders in schizophrenia will help identify more precisely the role of changes in specific circuitry in influencing different aspects of self-experience, and some progress in this direction has already been made (Raballo et al., 2011). Nevertheless, some task-dependent studies have attempted to establish which of the many altered neural circuits in schizophrenia may be of specific importance for altered self-reflection and sense of agency.

An influential recent task-dependent study has provided evidence for a possible anterior to posterior shift in activation of the CMS during a self-reflection paradigm where subjects were presented with positive and negative trait adjectives and asked to consider them in the context of describing self or other. Schizophrenia patients showed reduced activation in the right mPFC and increased activation in the bilateral middle/posterior cingulate gyri during self-reflection as well as reduced functional connectivity between the AC and middle/posterior cingulate (Holt et al., 2011). A subsequent study using a similar task has shown hyporesponsivity in the mPFC and hyperactivation in the precuneus during self-evaluation, both of which correlated with insight scores (Bedford et al., 2012). This further supports the view that reduced insight in schizophrenia may be related to impaired self-processing. Another study has found reduced activation in the AC in schizophrenia patients during self-monitoring of performance (Carter et al., 2001). Reduced functional connectivity between frontal and parietal components of the CMS has also been reported both during resting-state (associated with severity of hallucinations and delusions – Rotarska-Jagiela et al., 2010) and a working-memory task (Deserno et al., 2012). Additionally, increased activation during a self vs. other-reference task has been reported in the PCC and precuneus in the posterior CMS and the IPL and SMG in the posterior MNS as well as the post-central gyri (Shad et al., 2012).

A brain-wide resting-state functional connectivity analysis found that the greatest changes in schizophrenia occurred in these posterior regions of the CMS and MNS [notably involving the superior parietal gyrus and precuneus (CMS) and IPL, angular gyrus, and SMG (MNS)]. These CMS and MNS changes were associated with both positive (including delusions and suspiciousness/persecution) and negative symptoms, and had a high discriminative accuracy for distinguishing patients from healthy controls (Guo et al., 2012). While some reduced resting-state functional connectivity was also observed in the anterior CMS (AC and superior frontal gyrus) and MNS (IFG) regions, this was not related to symptom severity (Guo et al., 2012). Within the MNS, resting-state functional connectivity between the right IFG and the insula has been found to be decreased in schizophrenia patients (Moran et al., 2013), and increased right IFG/insula activation occurs during auditory hallucinations (Sommer et al., 2008).

There is also increasing evidence for altered functional connectivity between the MNS and CMS in schizophrenia with decreased resting-state connectivity between the IFG and PCC (Zhou et al., 2007) and between the insula and PCC (Moran et al., 2013). The main hub region affected in the resting-state analysis by Guo et al. (2012) was the IPL, in the posterior MNS, with its functional connections with posterior CMS regions (precuneus and superior parietal gyrus) strengthened, and those with the angular gyrus and SMG weakened. Thus schizophrenia patients may have decreased functional connectivity between frontal MNS regions and both anterior and posterior regions of the CMS but increased functional connectivity between posterior regions of both the MNS and CMS. Overall therefore, integration between the MNS and CMS self-processing systems would appear to be highly dysfunctional in schizophrenia patients.

Further evidence for an important role of altered IPL function in the posterior MNS in schizophrenia and self-processing has been provided by a number of other studies. An increased overlap of cortical maps in schizophrenia patients in medial frontal, medial parietal, IPL, and middle temporal cortex has been reported during implicit self vs. other voice distinctions, with altered IPL activity being positive correlated with positive symptom severity (Jardri et al., 2011). The IPL, angular gyrus, and SMG have also all been reported to be involved in action awareness, sense of agency, and self-recognition (Farrer et al., 2004, 2008; Torrey, 2007; Macuga and Frey, 2011). Further, repetitive transcranial stimulation of the IPL has been shown to interfere with self-other face discrimination in healthy subjects (Uddin et al., 2006) and parietal lobe epilepsy is associated with the occurrence of psychotic symptoms including delusions and hallucinations (Salanova et al., 1995). Finally, a magnetoencephalography study has reported reduced alpha and gamma band oscillations and phase-locking in the right inferior parietal cortex of schizophrenia patients during observation of biological motion, providing further evidence for altered mirror neuron properties in this region (Kato et al., 2011).

The potential contribution of reduced functional IHC in schizophrenia (Knöchel et al., 2012; Guo et al., 2013) to altered self-experience has yet to be fully established, although studies showing that connectivity between the hemispheres is important for a sense of agency, but not for self-recognition, are clearly suggestive (see Uddin, 2011). A recent resting-state study has provided evidence for a brain-wide reduction in functional connectivity involving symmetric regions in the two hemispheres, and including both anterior and posterior parts of the corpus callosum. Furthermore, this was correlated with severity of both positive and negative symptoms (Guo et al., 2013). For patients with psychiatric disorders associated with abnormalities of the callosum, schizophrenia is the most common (David et al., 1993) and other abnormalities in the corpus callosum are associated with severity of reality distortion in schizophrenia patients, although it would appear that smaller changes may be worse than larger ones in this respect (Whitford et al., 2010). A further interesting observation is that atypical cerebral lateralization, as evidenced in individuals who are right handed but left footed, is associated with schizotypal traits and an abnormal sense of agency (Asai et al., 2011).

**Figure 2:**
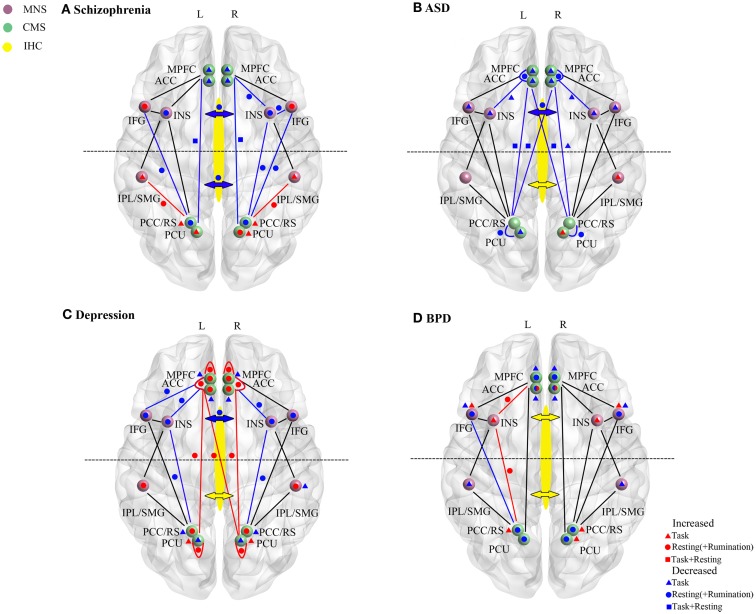
**Resting-state and task-dependent activity and functional connectivity changes in brain self-processing regions in (A) schizophrenia, (B) autism spectrum disorder (ASD), (C) depression, and (D) borderline personality disorder (BPD)**. Changes are summarized for the cortical midline (CMS) and mirror neuron (MNS) systems and for inter-hemispheric connectivity (IHC). Symbols denote overall increased (red) or decreased (blue) activity or functional connectivity changes reported in studies described in the main text. Where direction of changes seen in both resting-state (circle) and task (triangle) conditions are the same then this is indicated by a square. In all cases task data is only derived from studies reporting changes during self-processing tasks (including self-other discrimination, self-reference/evaluation; emotional empathy and recall of autobiographical information). For simplicity even where multiple studies in the text report the same direction of change in their findings only a single symbol is used. In the CMS increases or decreases are delineated in terms of the difference in the magnitude of the signal change compared to control subjects rather than the direction of change. For depression resting-state and rumination-induction studies are combined for simplicity since they always show changes in the same direction. Changes in frontal or middle/posterior functional connectivity via the corpus callosum and indicated by the anterior or posterior arrows (red, increase; blue, decrease). In some cases for BPD contradictory findings are indicated either within resting-state or task-dependent studies (i.e., for the AC and IFG). For abbreviations see Figure 1.

A summary of the main changes in self-processing neural networks in schizophrenia is provided in Figure 2A and Table 1. Overall there is evidence for disruption in both activity and functional connectivity involving the CMS, MNS, and IHC in schizophrenia in terms of resting-state activity and functional connectivity associated with symptom severity. This is supported by some task-dependent studies involving self-referential paradigms. In many cases neural changes in these self-processing systems are also correlated with severity of delusions and hallucinations and with poor insight. The general pattern of changes observed is of reduced resting-state activity and responses during self-referential tasks in frontal regions of the CMS and MNS and increased ones in posterior parietal regions of these two systems. There is also reduced functional connectivity between frontal and posterior components of the CMS both in resting-state and during self-referential tasks and between the anterior MNS and anterior/posterior CMS, although it is increased between the posterior regions of the MNS and CMS. Thus there appears to be a shift from a balanced and integrated CMS and MNS in terms of their anterior and posterior components toward and unbalanced and disconnected pattern in schizophrenia with a posterior hyperactivity bias. Furthermore, since both anterior and middle/posterior divisions of the corpus callosum show reduced structural and functional connectivity the resulting reduced inter-hemispheric connectivity between the anterior and posterior regions of the CMS and MNS may further exacerbate self-processing deficits.

**Table 1:** **Overall activity and functional connectivity changes in frontal and parietal cortical midline system regions involved in self-processing in schizophrenia, ASD, depression, and BPD**.

Disorder	Activity mPFC/AC	FC with IFG/INS	Frontal IH – FC/SC	Activity PCC/PCUN	FC with IPL	Parietal IH-FC/SC	Fronto-parietal FC
Schizophrenia	↓	↔	↓	↑ ↓	↑	↓	↓
ASD	↓	↓	↓	↑ ↓	↔	↔	↓
Depression	↑	↓	↓	↑ ↓	↔	↔	↑
BPD*	↓	↑	↔	↑ ↓	↔	↔	↔

AC, anterior/medial cingulate; ASD, autism spectrum disorder; BPD, borderline personality disorder; FC, functional connectivity; IFG, inferior frontal gyrus (frontal part of mirror neuron system); IH, inter-hemispheric; IPL, inferior parietal lobule (posterior part of mirror neuron system); PCC, posterior cingulate; PCUN, precuneus; SC, structural connectivity. *BPD changes are shown for patients without co-morbid disorders.

## Self-Processing in Autism

There is extensive evidence that self-other awareness is either impaired or delayed in terms of development in ASD, including self-recognition, body awareness, and sense of agency. Both clinical and research evidence has found that ASD patients have difficulties with sense of self and self-other confusion in terms of language use and make frequent pronoun reversal errors (I/me/you) (see Lyons and Fitzgerald, 2013). Individuals with ASD often have general postural and motor impairments as well as in action simulation, mimicry, and imitation (see Gallese et al., 2013), suggesting that similar to schizophrenia ASD patients may have impaired perception/action processing leading to problems in determining whether actions are self-generated or not. Indeed, there are a number of similarities between ASD and schizophrenia, including delusions, although while there are overlaps between the two disorders, and occasional co-morbidity, it is clear the severity of abnormal self-processing is much greater in schizophrenia (Fitzgerald, 2012).

As with schizophrenia resting-state and task-dependent fMRI and structural MRI studies have reported reduced connectivity involving long-distance connections but increased short-distance connectivity within frontal and temporal regions (see Uddin and Menon, 2009; Müller et al., 2011; Gallese et al., 2013; Lynch et al., 2013). It is argued that this results in a disruption in integrative processing between brain regions, and studies have shown altered connectivity in CMS and MNS, as well as in IHC, which may contribute to disordered self-processing (Anderson et al., 2011; Lyons and Fitzgerald, 2013).

In ASD, the CMS frontal (ventral mPFC and AC/medial cingulate) regions have been implicated in impairments in self-recognition (particularly self-face recognition – see Lyons and Fitzgerald, 2013), self-other discrimination in mentalizing tasks (Lombardo et al., 2010), and social behavior (Dapretto et al., 2006; Lynch et al., 2013). However, relatively few studies to date have specifically used task-dependent paradigms to investigate disordered self-processing in ASD. One notable exception to this is an elegant fMRI study using a visual imagery task, where adolescent ASD patients either watch others performing actions or imagine themselves performing the same action (Chiu et al., 2008). The results revealed a specific reduction in the response of the medial cingulate to self performed actions but not when observing those of others. In a second paradigm using a multi-round economic trust game the same deficit was observed in this medial cingulate region during self-decisions but not during those made by the partner in the game. The extent of reduced self-responses in the medial cingulate was also correlated with ASD symptom severity. In another task-dependent study using an introspective emotional awareness task, ASD patients were also found to show reduced activity in the left mPFC and right AC, although activity in the precuneus was decreased in the left hemisphere and increased in the right (Silani et al., 2008). Thus in the CMS, impaired anterior/medial cingulate function in ASD patients appears to be associated most consistently with self-processing dysfunction.

Resting-state functional connectivity within frontal (mPFC – AC; Vissers et al., 2012) and between frontal and parietal components of the CMS has been shown to be decreased in both adolescent and adult ADHD patients relative to controls (Monk et al., 2009; Assaf et al., 2010; Weng et al., 2010). However, this fronto-parietal functional connectivity increases significantly with age in healthy controls but does so to a lesser extent in ADHD patients (Wiggins et al., 2011). Reduced functional connectivity between frontal and parietal regions has also been shown in adult ASD patients in the context of an executive function task and was negatively correlated with Autism Diagnostic Observation Schedule (ADOS) scores (Just et al., 2007). A further recent study has reported increased resting-state PCC functional connectivity but decreased connectivity involving the precuneus, although not in relation to the frontal cortex (Lynch et al., 2013). Thus, similar to schizophrenia, there appear to be a number of connectivity and activity changes involving CMS parietal regions with a consistent finding being weakened functional connections between them and frontal regions of the CMS. Possibly the greater severity and delusional aspects of self-disorders in schizophrenia compared to ASD may reflect an even greater aberrant organizational shift toward posterior parietal regions and disconnection between them and frontal ones.

In view of motor and imitation impairments in ASD there has also been a recent focus on potential abnormalities in the MNS, although there is still considerable debate concerning the importance of the MNS role in this disorder (Dapretto et al., 2006; Rizzolatti and Fabbri-Destro, 2010; Enticott et al., 2012; Gallese et al., 2013). Studies to date have mainly highlighted reduced activation in frontal regions of the MNS (IFG), insula, and ventral pre-motor cortex (Dapretto et al., 2006; Silani et al., 2008; Uddin and Menon, 2009; Enticott et al., 2012) associated with severity of social dysfunction in ASD. Out of all of these MNS regions the most commonly reported finding is of reduced activity in the right anterior insula (see Uddin and Menon, 2009). There is also reduced functional connectivity between the bilateral insula and AC indicating a reduced interaction between frontal regions of the MNS and CMS in ASD (Vissers et al., 2012) similar to schizophrenia. However, in contrast to schizophrenia, altered responses in the posterior parietal components of the MNS (i.e., IPL and SMG), or their functional connectivity with the posterior part of the CMS (PCC and precuneus) have not generally been reported in ASD. Indeed, differences in the IFG responses, but not those of the IPL, have been found between adult ASD patients and controls during performance of a mental rotation task (Silk et al., 2006). However, a recent study has reported a different developmental trajectory for IPL responses during an emotional self-referencing task. Whereas control subjects showed a reduction in responses during adulthood compared to adolescence, ASD patients showed an increase. Indeed, IPL responses were negatively correlated with ASD symptom severity in adults although there were no overall differences between controls and ASD patients in either adolescence or adulthood (Schulte-Rüther et al., 2013). This finding suggests that age-associated compensatory changes may be occurring in ASD in the IPL as well as in resting-state functional connectivity involving the PCC (Lynch et al., 2013). Nevertheless, overall changes in parietal CMS and MNS regions in ASD appear to be less marked than those observed in schizophrenia and this may contribute to the greater severity of disturbed self-processing in this latter disorder.

While a number of studies have reported neural changes in ASD primarily in the right hemisphere, and this has led to the hypothesis that right hemisphere dysfunction is of greatest importance in this disorder (Lyons and Fitzgerald, 2013), there is nevertheless also evidence for involvement of altered inter-hemispheric connectivity. A recent structural and resting-state functional connectivity study found both significant reductions in corpus callosum volume and inter-hemispheric functional connectivity of the anterior insula and superior parietal lobule as well as sensorimotor cortex, fusiform gyrus, and superior temporal gyrus. Interestingly, only the functional connectivity changes were associated with ASD symptoms (Anderson et al., 2011). This study additionally reported a greater age-associated reduction in functional hemispheric connectivity in control subjects than in ASD patients, again suggesting possible age-associated compensatory changes in ASD. There may also be important links between structural changes in the corpus callosum and weakened functional connectivity between frontal and parietal regions of the CMS in ASD since these have been found to be correlated (Just et al., 2007). Another study reported 45% of children and 35% of adolescents with agenesis of the corpus callosum had scores on the Autism Spectrum Quotient above the autism-screening cut-off (Lau et al., 2012). A case study has also reported more severe impairment of self-referential behavior in an ASD patient with agenesis of the callosum (with damage primarily in the anterior and medial regions), although interestingly this was not associated with greater problems with appropriate first-person pronoun usage (Lombardo et al., 2012). Since the anterior part of the corpus callosum primarily connects between frontal brain regions (see Figure 1) this suggests that disturbed self-processing in ASD may be mainly contributed to by altered intra- and inter-hemisphere connections involving frontal regions.

A summary of the main changes in self-processing neural networks in ASD is provided in Figure 2B and Table 1. Overall there is consistent evidence for frontal regions of the CMS (mPFC and anterior and medial cingulate) and MNS (IFG and anterior insula) being hyporesponsive during self-processing in ASD. Reduced inter-hemispheric connectivity in ASD also seems mainly to occur in the anterior part of the corpus callosum linking frontal and anterior insula regions. While some evidence for either decreased or increased activity has been reported in the posterior CMS (precuneus) and MNS (IPL) this is less consistent. Age dependent compensatory changes seen in posterior parietal regions of the CMS and MNS, and in inter-hemispheric functional connectivity, may help to improve both balance and integration between anterior and posterior parts of the two systems. There is some evidence for improvements in severity of ASD symptoms with age, at least in high functioning patients (Happé and Charlton, 2012), and perhaps such compensatory changes may also help prevent the occurrence of more severe disturbances in self-processing in ASD such as those seen in schizophrenia.

## Self-Processing in Depression

Patients with major depression have a number of abnormalities in self-related processing, although these tend to be mostly in terms of increased self-focus, including excessive self-reflection (rumination) and associating themselves with negative emotions (see Northoff, 2007; Lemogne et al., 2012). Indeed, self-focus in depression has been shown to be a predictor of the likely re-occurrence of depressive episodes (see Nolen-Hoeksema et al., 2008). There have been a large number of resting-state and task-dependent studies reporting activity and functional connectivity changes in the DMN and regions controlling emotional and cognitive function in both first episode and treatment-resistant depression patients (see Wang et al., 2012). A recent study has also reported resting-state functional connectivity changes in the so-called “hate-circuit” comprising the superior frontal gyrus, insula, and putamen which may contribute to symptoms of “self-loathing” and reduced “self-esteem” which often occur in depressed patients (Tao et al., 2011). In this review we will focus primarily on changes in the CMS and MNS associated with rumination.

Neuroimaging studies have shown that frontal and parietal components of the DMN and CMS exhibit increased resting-state activity in depressed patients (see Wang et al., 2012), and that this reduces less than in controls when they view and appraise negative emotional stimuli (Sheline et al., 2009) or increases less in the mPFC and precuneus during self-focus (Grimm et al., 2009). It has been argued that the differences reported in the direction of altered mPFC activity in depression may reflect different interactions between mPFC and other regions in DMN and task-positive networks in event-related as opposed to block-based experimental paradigms (Lemogne et al., 2012). Indeed, another study has reported increased DMN dominance over task-positive regions (assessed by analysis of fronto-insular interactions) which correlated with higher maladaptive depressive rumination scores and reduced adaptive rumination ones (Hamilton et al., 2011).

During a task where depressed individuals were cued to ruminate they exhibited increased activity compared to controls in the CMS (AC and PCC) and in the posterior MNS (IPL), although in the anterior MNS (IFG) decreased activation occurred (Cooney et al., 2010). Importantly, a study has revealed that there may be a degree of dissociation between anterior and posterior CMS responses when individuals are cued to think about hopes and aspirations as opposed to duties and obligations (Johnson et al., 2009). Both depressed patients and healthy controls showed similar reduced activation in the anterior CMS (AC and medial frontal gyrus) when thinking about hopes and aspirations and increased activations in the posterior CMS (PCC and precuneus) when thinking about duties and obligations. In depressed patients anterior CMS responses to hopes and aspirations were negatively correlated with rumination scores whereas in the posterior regions they were positively correlated. In a second experiment in the same study depressed patients were found to exhibit reduced activation compared to controls in the anterior CMS during a self-evaluation condition where patients tend to focus more on negative self-referential thoughts. Negative self-referential thoughts tend to be perseverative in depressed patients and the study also found that they showed a reduced deactivation (i.e., actual activity levels were higher than in controls) responses in the posterior CMS (precuneus) during a distracter task. In both the anterior and posterior CMS regions activity during the distracter task was positively correlated with rumination scores. An anterior/posterior CMS dissociation in depression has also been shown in another study reporting reduced negative blood oxygen level dependent responses (NBRs) in the anterior CMS and increased ones in the posterior CMS during a self-relatedness task (Grimm et al., 2011). However, a previous study by the same group reported decreases in both anterior and posterior CMS regions using the same task (Grimm et al., 2009). This may reflect a medication effect since all the patients in the later study were medicated whereas in the earlier one they were not. Indeed, another study has shown that anti-depressant medications had a greater impact on functional connectivity changes in the posterior than in the anterior CMS (Li et al., 2013).

Another resting-state study has reported that functional connectivity between the mPFC and other DMN networks, as well as cognitive control and affective networks is increased (Sheline et al., 2010). Functional connectivity between the fronto-parietal CMS (AC and PCC) has also been found to increase during the resting-state, but not during a short-term memory task, and this was associated with rumination scores (Berman et al., 2011). Thus depression is consistently associated with increased functional connectivity between frontal and parietal regions of the CMS which distinguish it from schizophrenia and ASD where there is reduced connectivity. However, in line with anterior/posterior differences in activity changes in self-related tasks in depression a recent resting-state study has reported increased functional connectivity in the anterior CMS (mPFC and AC) which correlated positively with rumination scores, whereas in the posterior CMS (PCC and precuneus) reduced connectivity correlated negatively with over general autobiographical memory (Zhu et al., 2012). Another study has also reported increased functional connectivity within the frontal CMS (mainly mPFC) network and decreased in the posterior part (mainly precuneus) (Li et al., 2013). Additionally, this latter study found that successful treatment with anti-depressants only reversed functional connectivity changes in the posterior CMS.

Depressed patients also show decreased mimicry of happy face expressions (Schwartz et al., 1976) and reduced resting-state connectivity occurs in many components of the MNS including the IFG, insula, precentral gyrus SMG, and IPL (Tao et al., 2011). Reduced resting-state activity has been found in the insula in depression (Hamilton et al., 2011) and is associated with reduced interoceptive awareness (Wiebking et al., 2010). Further, there is some evidence for reduced resting-state functional connectivity between the insula and the AC and PCC in the CMS (see Sliz and Hayley, 2012) and between the IFG and AC (Wang et al., 2012). Possibly these weakened insula functional connections with both anterior and posterior CMS regions and between the IFG and the anterior CMS may contribute to the failure of depressed patients to disengage from negative thoughts and reflect a weakened influence of interoceptive cues and executive control over negative affect. Conversely, activity in the posterior part of the MNS (IPL) is increased during rumination (Cooney et al., 2010), suggesting that like the CMS there may be some differences between anterior and posterior components in depression. However overall, alterations in the MNS in depression which influence self-processing may be less influential than those in schizophrenia and ASD. Indeed, perhaps the reduced connectivity between the CMS and MNS in depression reflects a greater dominance of the CMS in depression and reduced potential conflict between CMS and MNS systems. This might help explain why depression does not result in a more severe self-processing dysfunction such as that in schizophrenia and ASD.

Inter-hemispheric connectivity is also reduced in depression and it has been proposed on the basis of TMS (Holtzheimer et al., 2001), electroencephalography (EEG) (Stewart et al., 2011), and fMRI (Kilgore et al., 2007) studies that an imbalance between left and right hemisphere activity may play an important role in contributing to this disorder. A diffusion tensor imaging study has found reduced fractional anisotropy in the anterior genu of the callosum which primarily connects between frontal cortical regions (Xu et al., 2013). However, to date no studies have attempted to associate IHC changes in depressed patients with self-focus or rumination, although it is interesting that as with ASD only connectivity between the frontal cortices may be impaired.

A summary of the main changes in self-processing neural networks in depression is provided in Figure 2C and Table 1. Overall the main pattern of changes involves increased resting-state activity in the anterior and posterior parts of the CMS and in the functional connectivity between them. During rumination patients also exhibit increased activity in these CMS regions as well as in the posterior MNS. During performance of self-reference tasks patients show either a reduced increase or decrease in activity in the CMS. However, while similar overall patterns of resting-state activity change usually occur in the anterior and posterior CMS several task-based studies have indicated a degree of dissociation between them with opposite directions of changes occurring during tasks, and also anti-depressant medication influencing posterior more than anterior changes in one case.

In the anterior MNS (insula and IFG) there is reduced resting-state activity in depressed patients and reduced functional connectivity with frontal (insula and IFG) and posterior (insula) regions of the CMS. There is also evidence for reduced inter-hemispheric connectivity involving frontal regions. Thus overall, depression is primarily associated with tonic hyperactivation in the CMS, contributed to by excessive rumination, and reduced activity changes in the same regions during self-referential tasks. These findings suggest an impaired ability to disengage from negative rumination during self-related tasks, perhaps contributing to an increased negative evaluation of self. This may also be contributed to by a reduced activity in the insula and its connections with both the frontal and posterior CMS, and between the IFG and anterior CMS, resulting in weakened control of negative affect due to impoverished interoceptive feedback and executive control.

The two core symptoms of depression are depressed mood and anhedonia. There is evidence for significant interactions between rumination and negative mood with rumination prolonging and deepening episodes of depression by promoting depressed mood. Rumination is also associated with suicidal ideation (Nolen-Hoeksema et al., 2008). The AC has been implicated both in negative mood and anhedonia and is increasingly regarded as a key region contributing to depression (Hamani et al., 2011; Pizzagalli, 2011; Treadway and Zald, 2011). While different functional connections of the AC are involved in terms of its influence on negative mood (amygdala) and anhedonia (fronto-striatal reward systems) compared to self-processing/rumination (insula and PCC/precuneus), there may be integration within the AC itself and this may help explain why this region is one of the most successful for the therapeutic application of deep brain stimulation in refractory depression (Hamani et al., 2011). Indeed, since rumination is considered to be highly predictive of the occurrence of depression it seems possible that rumination-induced changes in the AC in particular may promote the subsequent development of negative mood and anhedonia symptoms.

## Self-Processing in Borderline Personality Disorder

Borderline personality disorder patients have dysfunctional emotional regulation, impulse control, interpersonal relationships, and self-image/identity (Leichsenring et al., 2011). The diagnostic criteria for BPD under DSM-IV required that individuals have five out of nine symptoms, and only one of these: “Identify disturbance: notably and persistently unstable self-image or sense of self” specifically pertained to altered self-processing. However, in DSM-V diagnostic criteria for BPD specify that a person must have a significant impairment in personality functioning in relation to self. BPD patients also often have co-morbid post-traumatic stress disorder (PTSD), obsessive compulsive disorder, depression or dissociative disorder (DD) and this can make interpretation of neuroimaging findings more complex. Nevertheless, identity disturbance appears to be a core and distinctive component of BPD with patients expressing a sense of “self-fragmentation” and “falling apart” with four key features (role absorption: i.e., being absorbed in a single role or cause; painful incoherence: i.e., lack of a coherent subjective sense of self; inconsistency: i.e., objective evidence of incoherent behavior; lack of commitment: i.e., uncommitted to jobs or values) (Wilkinson-Ryan and Westen, 2000).

In contrast to schizophrenia, ASD, and depression there are less structural and functional neuroimaging studies in BPD and few of them have attempted to associate changes specifically with altered self-processing. Interpretational problems are also caused by the fact that BPD patients studied often have co-morbid PTSD, depression, obsessive compulsive disorder, or other personality disorders (Korzekwa et al., 2009; Leichsenring et al., 2011). However, studies contrasting BPD with DD can be particularly information because the latter primarily involves feelings that everything is unreal, of being disconnected from one’s body or feelings, and amnesia for autobiographical information (Korzekwa et al., 2009).

In terms of the CMS, the general pattern of findings from a variety of neuroimaging studies of BPD patients (mostly using positron emission tomography, PET) is for reduced size and resting-state activity in the mPFC and AC (see Lis et al., 2007; Korzekwa et al., 2009; Leichsenring et al., 2011; Wolf et al., 2011) although the mPFC is hyper-responsive to induced negative emotions and social exclusion, possibly reflecting reduced ability to exert emotional (Herpertz et al., 2001; Koenigsberg et al., 2009; Ruocco et al., 2010). However, one PET study has reported increased activity in female BPD patients bilaterally in the AC and in the right IFG although this may reflect co-morbidity with OCD/depression in many patients (Juengling et al., 2003). Another PET study with a small number of patients (*n* = 8) without co-morbidities has reported similar findings (Salavert et al., 2011) as well as hypometabolism in the PCC and precuneus. Thus the pattern of resting-state changes in the CMS is not entirely consistent and needs to be confirmed with appropriate account being taken of potential co-morbidity contributions.

During emotion and reward-related tasks a study has reported significantly reduced deactivation in the anterior/medial cingulate cortex and retrosplenial cortex (isthmus of the PCC) in BPD patients. Indeed, in the retrosplenial cortex tasks evoked activation rather than deactivation. The amount of deactivation in the retrosplenial cortex was positively correlated with the degree of personality organization (Doering et al., 2012). A recent meta-analysis of neural correlates of negative emotionality in BPD has also found reduced activation in the AC but increased in the insula and PCC (Ruocco et al., 2012). To date no study has reported altered functional connectivity between the frontal and parietal regions of the CMS in BPD, which is perhaps surprising given similarities with schizophrenia and ASD in terms of identity disturbances.

Interestingly, the right parietal cortex (PCC and precuneus) has been reported to be reduced in size in BPD resulting in a greater left to right asymmetry (Irle et al., 2005). However, another study which found reductions in both AC and PCC gray matter in BPD, but corresponding increases in white matter, showed that PCC changes only occurred in patients with co-morbid schizotypal personality disorder (Hazlett et al., 2005). Indeed, Irle et al. (2005) also reported a positive correlation between gray matter volume in the parietal cortex and psychotic symptoms in their cohort of BPD subjects and in a subsequent study found that volume changes in the superior parietal cortex were correlated with dissociative symptoms (Irle et al., 2007). Thus CMS parietal changes in BPD, as in schizophrenia and ASD, may be particularly associated with delusional psychotic symptoms contributing to a disordered sense of self.

Some structural and activity/functional connectivity changes have been found in both frontal and parietal components of the MNS in BPD, although no problems with facial expression/gesture imitation have been reported (Lis et al., 2007; Korzekwa et al., 2009; Leichsenring et al., 2011). Similar to the other disorders, resting-state functional connectivity changes involving the IFG and the insula have been reported (Wolf et al., 2011). Increased functional connectivity between the left insula and both the anterior (mPFC) and posterior (PCC/precuneus) CMS has been found which correlated with scores on the dissociative tension scale (Wolf et al., 2011). Furthermore, increased bilateral activation in the insula of BPD patients has been reported during an emotional empathy task (Dziobek et al., 2011). Another study has investigated effects of recalling autobiographical memories of resolved compared with unresolved life events in BPD patients (Beblo et al., 2006). This also found increased bilateral insula activation as well as in the IFG when contrasting the difference between responses to unresolved vs. resolved life events in patients vs. controls. However, decreased IFG activity has also been reported in BPD patients during a theory of mind task involving emotional attributions (Mier et al., 2013) and in response to induced aggression (New et al., 2009). Finally some evidence for decreased functional connectivity between the left PCC and the IFG (i.e., the anterior MNS) has been found during pain processing in BPD patients (Kluetsch et al., 2012). Only one study has found evidence for reduced activation of the posterior MNS (IPS) in BPD patients during a task involving distancing from negative emotion pictures (Koenigsberg et al., 2009).

Overall therefore, there is increasing evidence for hyperactivation in the insula in the frontal MNS regions which may be particularly associated with altered self-processing in BPD, although there also appear a number of alterations involving the IFG. Interestingly BPD is the only one of the four disorders reviewed here where insula activity and connectivity with the CMS is increased since in schizophrenia, ASD, and depression it is decreased. Also changes in the insula connectivity changes in BPD have only been reported in the left hemisphere. As with the CMS however, more studies are needed to clarify the precise changes occurring in the MNS, as well as its interactions with the CMS, that are specifically associated with identity disturbance in BPD.

While one study has reported a reduced size of the isthmus of the corpus callosum (which connects parietal and temporal lobes) in BPD patients, they had co-morbidity with PTSD (Rüsch et al., 2007), and another study failed to find any evidence for altered corpus callosum structure in first episode BPD patients (Walterfang et al., 2010). A more focused study using diffusion tensor imaging has reported a specific reduction in inter-hemispheric fibers connecting the AC in BPD, although this too involved patients with PTSD co-morbidity (Rüsch et al., 2010). Thus at this point it seems unlikely that altered IHC contributes specifically to disordered self-processing in BPD itself.

A summary of the main changes in self-processing neural networks in BPD is provided in Figure 2D and Table 1. While some degree of caution is required in drawing overall conclusions about the precise pattern of changes occurring, due to the relatively small number of studies carried out and co-morbidity problems, studies have found altered activity and task-responsivity in frontal and posterior CMS regions (particularly the AC), although the direction of observed changes is somewhat inconsistent. On the other hand there is increasing evidence for hyper-responsivity and functional connectivity in the insula in the MNS, while the IFG generally shows either reduced or increased activity. There is also some evidence for reduced activity in the IPL. While there is evidence for structural and some functional changes in parietal CMS regions (PCC/retrosplenial cortex and precuneus) at this point it is probable that these may be contributed to by schizoid co-morbidity rather than BPD *per se*. Co-morbidity issues may also apply to reported changes in IHC.

Thus at this point the main changes observed in BPD itself would appear to reside in frontal regions of the CMS and MNS self-processing systems, with a distinguishing feature being left hemisphere dominated increased insula activity and functional connectivity with the CMS. A common feature between BPD and schizophrenia is also reduced connectivity between the IFG and posterior CMS which may also reflect the importance of reduced integration between anterior and posterior networks in self-processing dysfunction. Given the roles of the AC and mPFC in both control of affective responses and impulsivity, both of which show impairments in BPD (Leichsenring et al., 2011), it could be speculated that there may be potential overlap between disordered self-identity and affective and impulse control in these regions. Interestingly, there is a significant correlation between identity disturbance and affective instability but not with impulsive-aggression in BPD (Koenigsberg et al., 2001), which suggests a possible interaction between disordered self-processing and affective control in BPD. However, fronto-amygdala pathways are of most importance in the latter, and indeed amygdala hyper-responsivity to negative emotional stimuli is a key feature of BPD (Lis et al., 2007; Korzekwa et al., 2009; Leichsenring et al., 2011). On the other hand fronto-amygdala pathways do not appear to play a significant role in self-processing.

## Conclusion and Future Directions

Overall it is clear that disordered self-processing in schizophrenia, ASD, depression, and BPD is associated with alterations in the CMS although the patterns of changes are somewhat different. A summary of these changes in Figure 2 and Table 1 shows that in schizophrenia, ASD, and BPD there is general evidence for reduced activity in the mPFC whereas in depression it is increased. The same pattern is seen for the AC except in BPD where conflicting findings have been reported. On the other hand, in the posterior parietal cortex part of the CMS activity changes in all four disorders are more varied, although with schizophrenia and depression showing a more consistent pattern of increase. A key feature when comparing the disorders is that resting-state functional connectivity between frontal and parietal regions of the CMS is decreased in schizophrenia and in ASD patients but increased in depression and unchanged in BPD. In schizophrenia, functional connectivity between frontal MNS regions (insula and IFG) and the posterior CMS is also reduced and thus in this disorder there is an almost complete disconnection between anterior and posterior parts of the CMS and MNS. Another unique feature of schizophrenia is that functional connections between the posterior parts of the CMS and MNS are increased. Thus, there is support for the view that there is a widespread functional shift from anterior frontal to posterior parietal parts of both the CMS and MNS in schizophrenia which may contribute to the severity of self-processing dysfunction in this disorder. On the other hand in the two other disorders with identity disturbance, ASD and BPD, neither has dysfunction in both the anterior and posterior CMS and MNS systems. Weakened functional connectivity in ASD is mainly restricted to the CMS, although with some reduced connectivity between anterior parts of the two systems, and in BPD only altered functional connectivity between the anterior and posterior MNS occurs. While functional connections between both of the anterior and posterior components of the CMS and MNS systems are altered in depression they show a degree of balance, with increases in the CMS and decreases in the MNS. This suggests that integration between processing in anterior and posterior regions is maintained, although with a bias toward CMS dominance which may act to promote excessive rumination and negative mood but without identity disturbance. Interestingly task-based studies in depressed patients suggest a degree of dissociation between responses in anterior and posterior parts of the CMS and also differential sensitivity to anti-depressant medication. This further underlines the importance of maintaining integrated functioning between frontal and posterior networks for optimal self-processing and suggests that different neurochemical signaling systems may be involved in regulating functional changes in each of them. Thus overall the patterns of changes observed in the four different disorders support a general hypothesis that self-identity disturbances in particular may primarily result from any breakdown in integrated interactions between the frontal and posterior components of both the CMS and the MNS. The severity of identity disturbance may depend on the extent to which disconnection of frontal and posterior components of both the CMS and MNS occurs.

There is increasing evidence that connectivity between the frontal and posterior parts of the CMS is disrupted during anesthesia or brain-damaged induced loss of consciousness (Boveroux et al., 2010; Vanhaudenhuyse et al., 2010) and that DMN function (Anticevic et al., 2012) and brain functional connectivity (Stephan et al., 2009) is influenced by NMDA-receptor signaling. As such, reduced connectivity in psychiatric disorders may produce disorders of both self and consciousness, which is particularly relevant in relation to schizophrenia (Sass and Parnas, 2003) where functional dysconnectivity has been associated with aberrant NMDA-receptor signaling (Stephan et al., 2009). NMDA-receptor antagonists, such as the dissociative ketamine, can elicit schizophrenia symptoms in healthy subjects and disrupt task-dependent fronto-parietal functional connectivity (Anticevic et al., 2012). Treatments with NMDA-receptor agonists have also produced some positive results in reducing schizophrenia symptoms (Tsai and Lin, 2010) as well as in ASD (Posey et al., 2004). The rapid anti-depressant effects of ketamine might be potentially explained by a reduction in the abnormally increased functional connectivity between anterior and posterior regions of the CMS in depression. This has received some recent support from a study showing that ketamine does indeed reduce functional connectivity between the AC/mPFC and PCC in the CMS (Scheidegger et al., 2012). Impairments in NMDA-receptor function are also thought to play a role in BPD (Grosjean and Tsai, 2007). Clearly this is an important focus for future research and it will also be interesting to investigate the specific effects of NMDA-receptor treatments on self-processing dysfunction in these psychiatric disorders.

At this point the specific contribution of the MNS to disordered self-processing in schizophrenia, ASD, depression, and BPD is still difficult to assess, and caution is obviously required in making any broad assumptions that altered functioning in any MNS region relates to dysfunctional mirror neuron properties, since these regions are involved in other cognitive and emotional functions. Given the presence of impaired mimicry and other motor functions in schizophrenia and ASD, it is reasonable to suggest that changes in frontal (IFG and insula) and parietal (IPL/SMG) MNS regions and their links to pre-motor and motor cortices may contribute both to an impaired sense of physical self and self-other action attribution, particularly as a result of reduced temporal contiguity between perception/intention and action processing. Furthermore, enhanced functional connectivity between the parietal MNS and CMS in schizophrenia may serve to exacerbate the severity of symptoms of physical self-disorder, and perhaps help extend them more pervasively into cognitive and emotional domains. In depression, only relatively minor effects on face emotion mimicry have been reported and it is therefore unlikely that these reflect reduced temporal contiguity in perception-action processing as opposed to a general reduction in attention and responsivity to external stimuli. In this respect it is interesting that unlike the mixed pattern of changes in schizophrenia and BPD functional connectivity between the MNS and CMS is consistently reduced in depression. As already mentioned, this may perhaps reflect an increased dominance of the CMS over MNS self-processing systems in depression but without resulting in significant conflict between them which may be occurring in schizophrenia and BPD as a result of differential patterns of change.

Changes in IHC are also most evident in schizophrenia and appear to involve reduced connections between both frontal and parietal regions via the corpus callosum. Since the main aspect of self-processing that is affected by callosal damage and atypical cerebral lateralization is a sense of agency (Asai et al., 2011; Uddin, 2011), one can speculate that misattribution of agency in schizophrenia may be at least partly a consequence of reduced IHC. However, this remains to be established. In ASD, reduced connectivity via anterior/medial regions of the corpus callosum, which primarily link frontal areas, may also serve to further increase disordered self-processing resulting from frontal dysfunction, and it is interesting that many individuals with callosal agenesis have high scores on the Autism Spectrum Quotient. However, once again the precise contribution of changes to IHC on altered self-processing *per se* require further studies. With BPD on the other hand reported reductions in IHC may only occur in patients with co-morbid ADHD and so are less likely to contribute to sense of identity problems in this disorder.

From this review it can be seen that while some progress has been made toward identifying the neural correlates of abnormal self-processing in different psychiatric disorders, there is an urgent need for more future studies to include better assessments of specific aspects of self-processing which will permit more precise functional correlations between neural and behavioral changes to be made. Overall, studies serve to confirm the importance of the CMS in self-processing that is being increasingly established by studies using healthy subjects, although they also indicate that involvement of the MNS and IHC, and interactions between them and the CMS may be of key importance. It would seem therefore that while many different neural systems contribute to dysfunctional self-processing in human psychiatric disorders, some common and differential patterns of altered activity and functional connections involving the CMS, MNS, and IHC systems are beginning to emerge. The precise identification of which network components contribute to specific aspects of self-disorders and how they relate to more general impairments in cognitive, social, and emotional functioning is an important future challenge in both neuroscience and psychiatry.

## Conflict of Interest Statement

The authors declare that the research was conducted in the absence of any commercial or financial relationships that could be construed as a potential conflict of interest.

## References

[B1] Anderson J. S. Druzgal T. J. Froehlich A. DuBray M. B. Lange N. Alexander A. L. (2011). Decreased interhemispheric connectivity in autism. Cereb. Cortex 21, 1134–1146 10.1093/cercor/bhq190 20943668 PMC3077433

[B2] Anticevic A. Gancsos M. Murray J. D. Repovs G. Driesen N. R. Ennis D. J. (2012). NMDA receptor function in large-scale anticorrelated neural systems with implications for cognition and schizophrenia. Proc. Natl. Acad. Sci. U.S.A. 109, 16720–16725 10.1073/pnas.1208494109 23012427 PMC3478611

[B3] Asai T. Sugimori E. Tanno Y. (2011). A psychometric approach to the relationship between hand-foot preference and auditory hallucinations in the general population: atypical lateralization may cause an abnormal sense of agency. Psychiatry Res. 189, 220–227 10.1016/j.psychres.2011.02.014 21439651

[B4] Assaf M. Jagannathan K. Calhoun V. D. Miller L. Stevens M. C. Sahl R. (2010). Abnormal functional connectivity of default mode sub-networks in autism spectrum disorder patients. Neuroimage 53, 247–256 10.1016/j.neuroimage.2010.05.067 20621638 PMC3058935

[B5] Beblo T. Driessen T. Mertens M. Wingenfeld K. Piefke M. Rullkoetter N. (2006). Functional MRI correlates of the recall of unresolved life events in borderline personality disorder. Psychol. Med. 36, 845–856 10.1017/S0033291706007227 16704749

[B6] Bedford N. J. Surguladze S. Giampietro V. Brammer M. J. David A. S. (2012). Self-evaluation in schizophrenia: an fMRI study with implications for the understanding of insight. BMC Psychiatry 12:106 10.1186/1471-244X-12-106 22876974 PMC3527271

[B7] Berman M. G. Peltier S. Nee D. E. Kross E. Deldin P. J. Jonides J. (2011). Depression, rumination and the default network. Soc. Cogn. Affect. Neurosci. 6, 548–555 10.1093/scan/nsq080 20855296 PMC3190207

[B8] Blakemore S. J. Wolpert D. Frith C. (2000). Why can’t you tickle yourself? Neuroreport 11, R11–R16 10.1097/00001756-200008030-00002 10943682

[B9] Boveroux P. Vanhaudenhuyse A. Bruno M.-A. Noirhomme Q. Lauwick S. Luxen A. (2010). Breakdown of within- and between-network resting state functional magnetic resonance imaging connectivity during propofol-induced loss of consciousness. Anesthesiology 113, 1038–1053 10.1097/ALN.0b013e3181f697f5 20885292

[B10] Brown W. S. Paul L. K. (2000). Cognitive and psychosocial deficits in agenesis of the corpus callosum with normal intelligence. Cogn. Neuropsychiatry 5, 135–157 10.1080/135468000395781

[B11] Carter C. S. MacDonald A. W. Ross L. L. Stenger A. (2001). Anterior cingulate cortex activity and impaired self-monitoring of performance in patients with schizophrenia: an event-related fMRI study. Am. J. Psychiatry 158, 1423–1428 10.1176/appi.ajp.158.9.1423 11532726

[B12] Cattaneo L. Rizzolatti G. (2009). The mirror neuron system. Arch. Neurol. 66, 557–560 10.1001/archneurol.2009.41 19433654

[B13] Chiu P. H. Kayali M. A. Kishida K. T. Tomlin D. Klinger L. G. Klinger M. R. (2008). Self responses along cingulate cortex reveal quantitative neural phenotype for high functioning autism. Neuron 57, 463–473 10.1016/j.neuron.2007.12.020 18255038 PMC4512741

[B14] Choi H. Kubicki M. Whitford T. J. Alvarado J. L. Terry D. P. Niznikiewicz M. (2011). Diffusion tensor imaging of anterior commissural fibers in patients with schizophrenia. Schizophr. Res. 130, 78–85 10.1016/j.schres.2011.04.016 21561738 PMC3745276

[B15] Christoff K. Cosmelli F. Legrand D. (2011). Thompson E. Specifying the sense of self for neuroscience. Trends Cogn. Sci. (Regul. Ed.) 15, 104–112 10.1016/j.tics.2011.01.001 21288760

[B16] Clinton S. M. Meador-Woodruff J. H. (2004). Thalamic dysfunction in schizophrenia: neurochemical, neurophysiological, and in vivo imaging abnormalities. Schizophr. Res. 69, 237–253 10.1016/j.schres.2003.09.017 15469196

[B17] Cooney R. E. Joormann J. Eugéne F. Dennis E. L. Gotlib I. H. (2010). Neural correlates of rumination in depression. Cogn. Affect. Behav. Neurosci. 10, 470–478 10.3758/CABN.10.4.470 21098808 PMC4476645

[B18] Crow T. J. (1998). Schizophrenia as a transcallosal misconnection syndrome. Schizophr. Res. 30, 111–114 10.1016/S0920-9964(97)00139-4 9549773

[B19] Dapretto M. Davies M. S. Pfeifer J. H. Scott A. A. Sigman M. Bookheimer S. Y. (2006). Understanding emotions in others: mirror neuron dysfunction in children with autism spectrum disorders. Nat. Neurosci. 9, 28–30 10.1038/nn1611 16327784 PMC3713227

[B20] David A. S. Wacharasindhu A. Lishman W. A. (1993). Severe psychiatric disturbance and abnormalities of the corpus callosum: review and case series. J. Neurol. Neurosurg. Psychiatr. 56, 85–93 10.1136/jnnp.56.1.85 8429328 PMC1014772

[B21] Deserno L. Sterzer P. Wüstenberg T. Heinz A. Schlagenhauf F. (2012). Reduced prefrontal-parietal effective connectivity and working memory deficits in schizophrenia. J. Neurosci. 32, 12–20 10.1523/JNEUROSCI.3405-11.2012 22219266 PMC6621317

[B22] Doering S. Enzi B. Faber C. Hinrichs J. Bahmer J. Northoff G. (2012). Personality functioning and the cortical midline structures – an exploratory fMRI study. PLoS ONE 7:e49956 10.1371/journal.pone.0049956 23189175 PMC3506600

[B23] Dziobek I. Preiβter S. Grozdanovic Z. Heuser I. Heekeren H. R. Roepke S. (2011). Neuronal correlates of altered empathy and social cognition in borderline personality disorder. Neuroimage 57, 539–548 10.1016/j.neuroimage.2011.05.005 21586330

[B24] Enticott P. G. Kennedy H. A. Rhinehart N. J. Tonge B. J. Bradshaw J. L. Taffe J. R. (2012). Mirror neuron activity associated with social impairments but not age in autism spectrum disorder. Biol. Psychiatry 71, 427–433 10.1016/j.biopsych.2011.09.001 21974786

[B25] Farrer C. Franck N. Frith C. D. Decety J. Georgieff N. d’Amato T. (2004). Neural correlates of action attribution in schizophrenia. Psychiatry Res. 131, 31–44 10.1016/j.pscychresns.2004.02.004 15246453

[B26] Farrer C. Frey S. H. Van Horn J. D. Tunik E. Turk D. Inati S. (2008). The angular gyrus computes action awareness representations. Cereb. Cortex 18, 254–261 10.1093/cercor/bhm050 17490989

[B27] Ferri F. Frassinetti F. Ardizzi M. Costatini M. Gallese V. (2012a). A sensorimotor network for the bodily self. J. Cogn. Neurosci. 24, 1584–1595 10.1162/jocn_a_00230 22452562

[B28] Ferri F. Frassinetti F. Mastrangelo F. Salone A. Maria Ferro F. Gallese V. (2012b). Bodily self and schizophrenia: the loss of implicit self-body knowledge. Conscious. Cogn. Available at: http://dx.doi.org/10.1016/j.concog.2012.05.001 10.1016/j.concog.2012.05.001 22673373

[B29] Fitzgerald M. (2012). Schizophrenia and autism/Asperger’s syndrome: overlap and difference. Clin. Neuropsychiatry 9, 171–176

[B30] Fox M. D. Synder A. Z. Vincent J. L. Corbetta M. Van Essen D. C. Raicle M. E. (2005). The human brain is intrinsically organized into dynamic, anticorrelated functional networks. Proc. Natl. Acad. Sci. U.S.A. 102, 9673–9678 10.1073/pnas.0504136102 15976020 PMC1157105

[B31] Friston K. J. Frith C. D. (1995). Schizophrenia: a disconnection syndrome? Clin. Neurosci. 3, 89–97 7583624

[B32] Frith C. D. Blakemore S. J. Wolpert D. M. (2000). Abnormalities in the awareness and control of action. Philos. Trans. R. Soc. Lond. B Biol. Sci. 355, 1771–1788 10.1098/rstb.2000.0734 11205340 PMC1692910

[B33] Gallese V. Rochat M. J. Berchio C. (2013). The mirror mechanism and its potential role in autism spectrum disorder. Dev. Med. Child Neurol. 55, 15–22 10.111/j.1469-8749.2012.04398.x 22924341

[B34] Garrity A. G. Pearlson G. D. McKiernan K. Lloyd D. Kiehl K. A. Calhoun V. D. (2007). Aberrant “default mode” functional connectivity in schizophrenia. Am. J. Psychiatry 164, 450–457 10.1176/appi.ajp.164.3.450 17329470

[B35] Gillihan S. J. Farah M. J. (2005). Is self special? A critical review of evidence from experimental psychology and cognitive neuroscience. Psychol. Bull. 131, 76–97 10.1037/0033-2909.131.1.76 15631554

[B36] Grimm S. Ernst J. Boesiger P. Schuepbach D. Boeker H. Northoff G. (2011). Reduced negative BOLD responses in the default-mode network and increased self-focus in depression. World J. Biol. Psychiatry 12, 627–637 10.3109/15622975.2010.545145 21247256

[B37] Grimm S. Ernst J. Boesiger P. Schuepbach D. Hell D. Boeker H. (2009). Increased self-focus in major depressive disorder is related to neural abnormalities in subcortical-cortical midline structures. Hum. Brain Mapp. 30, 2617–2627 10.1002/hbm.20693 19117277 PMC6870821

[B38] Grosjean B. Tsai G. E. (2007). NMDA neurotransmission as a critical mediator of borderline personality disorder. J. Psychiatry Neurosci. 32, 103–115 17353939 PMC1810584

[B39] Guo S. X. Kendrick K. M. Yu R. Tseng W. Y. Feng J. (2012). Key functional circuitry altered in schizophrenia involves parietal regions associated with sense of self. Hum. Brain Mapp. 10.1002/hbm.22162 23008170 PMC6869177

[B40] Guo S. X. Kendrick K. M. Zhang J. Yu R. Liu Z. Feng J. (2013). Brain-wide functional inter-hemispheric disconnectivity is a potential biomarker for schizophrenia and distinguishes it from depression. Neuroimage 2, 818–826 10.1016/j.nicl.2013.06.008 PMC3777798 24179833

[B41] Gusnard D. A. Akbudak E. Shulman G. L. Raichle M. E. (2001). Medial prefrontal cortex and self-referential mental activity: relation to a default mode of brain function. Proc. Natl. Acad. Sci. U.S.A. 98, 4259–4264 10.1073/pnas.071043098 11259662 PMC31213

[B42] Haker H. Rössler W. (2009). Empathy in schizophrenia: impaired resonance. Eur. Arch. Psychiatry Clin. Neurosci. 259, 352–361 10.1007/s00406-009-0007-3 19377866

[B43] Hamani C. Mayberg H. Stone S. Laxton A. Haber S. Lozano A. M. (2011). The subcallosal cingulate gyrus in the context of major depression. Biol. Psychiatry 69, 301–308 10.1016/j.biopsych.2010.09.034 21145043

[B44] Hamilton J. P. Furman D. J. Chang C. Thomason M. E. Dennis E. Gotlib I. H. (2011). Default-mode and task-positive network activity in major depressive disorder: implications for maladaptive rumination. Biol. Psychiatry 70, 327–333 10.1016/j.biopsych.2011.02.003 21459364 PMC3144981

[B45] Happé F. Charlton R. A. (2012). Aging in Autism spectrum disorders: a mini-review. Gerontology 58, 70–78 10.1159/000329720 21865667

[B46] Hazlett E. A. New A. S. Newmark R. Haznedar M. M. Lo J. N. Speiser L. J. (2005). Reduced anterior and posterior cingulate gray matter in borderline personality disorder. Biol. Psychiatry 58, 614–623 10.1016/j.biopsych.2005.04.029 15993861

[B47] Herpertz S. C. Dietrich T. M. Wenning B. Krings T. Erberich S. G. Willmes K. (2001). Evidence of abnormal amygdala functioning in borderline personality disorder: a functional MRI study. Biol. Psychiatry 50, 292–298 10.1016/S0006-3223(01)01075-7 11522264

[B48] Holt D. J. Cassidy B. S. Andrews-Hanna J. R. Lee S. M. Coombs G. Goff D. C. (2011). An anterior-to-posterior shift in midline cortical activity in schizophrenia during self-reflection. Biol. Psychiatry 69, 415–423 10.1016/j.biopsych.2010.10.003 21144498 PMC3740539

[B49] Holtzheimer P. E. III Russo J. Avery D. H. (2001). A meta-analysis of repetitive transcranial magnetic stimulation in the treatment of depression. Psychopharmacol. Bull. 35, 149–169 12397863

[B50] Huang X. Q. Lui S. Deng W. Chan R. C. Wu Q. Z. Jiang L. J. (2010). Localization of cerebral functional deficits in treatment-naive, first-episode schizophrenia using resting-state fMRI. Neuroimage 49, 2901–2906 10.1016/j.neuroimage.2009.11.072 19963069

[B51] Irle E. Lange C. Sachsse U. (2005). Reduced size and abnormal asymmetry of parietal cortex in women with borderline personality disorder. Biol. Psychiatry 57, 173–182 10.1016/j.biopsych.2004.10.004 15652877

[B52] Irle E. Lange C. Weniger G. Sachsse U. (2007). Size abnormalities of the superior parietal cortices are related to dissociation in borderline personality disorder. Psychiatry Res. 156, 139–149 10.1016/j.pscychresns.2007.01.007 17826965

[B53] Jardri R. Pins D. Lafargue G. Very E. Ameller A. Delmaire C. (2011). Increased overlap between the brain areas involved in self-other distinction in schizophrenia. PLoS ONE 6:e17500 10.1371/journal.pone.0017500 21408008 PMC3052363

[B54] Jeannerod M. (2003). The mechanism of self-recognition in humans. Behav. Brain Res. 142, 1–15 10.1016/S0166-4328(02)00384-4 12798261

[B55] Johnson M. K. Nolen-Hoeksema S. Mitchell K. J. Levin Y. (2009). Medial cortex activity, self-reflection and depression. Soc. Cogn. Affect. Neurosci. 4, 313–327 10.1093/scan/nsp022 19620180 PMC2799950

[B56] Juengling F. D. Schmahl C. Hesslinger B. Ebert D. Bremner J. D. Gostomzyk J. (2003). Positron emission tomography in female patients with borderline personality disorder. J. Psychiatr. Res. 37, 109–115 10.1016/S0022-3956(02)00084-5 12842164

[B57] Just M. A. Cherkassky V. L. Keller T. A. Kana R. K. Minshew N. J. (2007). Functional and anatomical cortical underconnectivity in Autism: evidence from an fMRI study of an executive function task and corpus callosum morphometry. Cereb. Cortex 17, 951–961 10.1093/cercor/bhl006 16772313 PMC4500121

[B58] Kato Y. Muramatsu M. Kato M. Shibukawa Y. Shintani M. Mimura M. (2011). Magnetoencephalography study of right parietal lobe dysfunction of the evoked mirror neuron system in antipsychotic-free schizophrenia. PLoS ONE 6:e28087 10.1371/journal.pone.0028087 22132217 PMC3222679

[B59] Kilgore W. D. Gruber S. A. Yurgelun-Todd D. A. (2007). Depressed mood and lateralized prefrontal activity during a Stroop task in adolescent children. Neurosci. Lett. 416, 43–48 10.1016/j.neulet.2007.01.081 17350756 PMC1964792

[B60] Kircher T. T. J. David A. (eds). (2003). The Self in Neuroscience and Psychiatry. Cambridge: Cambridge University Press

[B61] Kluetsch R. C. Schmahl C. Niedtfeld I. Densmore M. Calhoun V. D. Daniels J. (2012). Alterations in default mode network connectivity during pain processing in borderline personality disorder. Arch. Gen. Psychiatry 69, 993–1002 22637967 10.1001/archgenpsychiatry.2012.476 PMC4429518

[B62] Knöchel C. Oertel-Knöchel V. Schönmeyer R. Rotarska-Jagiela A. van den Ven V. Prvulovic D. (2012). Interhemispheric hypoconnectivity in schizophrenia: fiber integrity and volume differences of the corpus callosum in patients and unaffected relatives. Neuroimage 50, 926–934 10.1016/j.neuroimage.2011.07.088 21964509

[B63] Koenigsberg H. W. Harvey P. D. Mitropolou V. New A. S. Goodman M. Silverman J. (2001). Are the interpersonal and identity disturbances in the borderline personality disorder criteria linked to traits of affective instability and impulsivity? J. Pers. Disord. 15, 358–370 10.1521/pedi.15.4.358.19181 11556702

[B64] Koenigsberg H. W. Siever L. J. Lee H. Pizzarello S. New A. S. Goodman M. (2009). Neural correlates of emotion processing in borderline personality disorder. Psychiatry Res. 172, 192–199 10.1016/j.pscychresns.2008.07.010 19394205 PMC4153735

[B65] Korzekwa M. I. Dell P. F. Pain C. (2009). Dissociation and borderline personality disorder: an update for clinicians. Curr. Psychiatry Rep. 11, 82–88 10.1007/s11920-009-0013-1 19187714

[B66] Krawitz A. Braver T. S. Barch D. M. Brown J. W. (2011). Impaired error-likelihood in medial prefrontal cortex in schizophrenia. Neuroimage 54, 1506–1517 10.1016/j.neuroimage.2010.09.027 20851194 PMC2997134

[B67] Lau Y. C. Hinkley L. B. M. Bukshpun P. Strominger Z. A. Wakahiro M. L. J. Baron-Cohen S. (2012). Autism traits in individuals with agenesis of the corpus callosum. J. Autism Dev. Disord. 43, 1106–1118 10.1007/s10803-012-1653-2 23054201 PMC3625480

[B68] Legrand D. Ruby P. (2009). What is self-specific? Theoretical investigation and critical review of neuroimaging results. Psychol. Rev. 116, 252–282 10.1037/a0014172 19159156

[B69] Leichsenring F. Leibing E. Kruse J. New A. S. Leweke F. (2011). Borderline personality disorder. Lancet 377, 74–84 10.1016/S0140-6736(10)61422-5 21195251

[B70] Lemogne C. Delaveau P. Freton M. Guionnet S. Fossati P. (2012). Medial prefrontal cortex and the self in major depression. J. Affect. Disord. 136, e1–e11 10.1016/j.jad.2010.11.034 21185083

[B71] Li B. Liu L. Friston K. J. Shen H. Wang L. Zeng L.-L. (2013). A treatment-resistant default mode subnetwork in major depression. Biol. Psychiatry 74, 48–54 10.1016/j.biopsych.2012.11.007 23273724

[B72] Lis E. Greenfield B. Henry M. Guilé J. M. Dougherty G. (2007). Neuroimaging and genetics of borderline personality disorder: a review. J. Psychiatry Neurosci. 32, 162–173 17476363 PMC1863557

[B73] Lombardo M. V. Chakrabarti B. Bullmore E. T. Sadek S. A. Pasco G. Wheelwright S. J. (2010). Atypical neural self-representation in autism. Brain 133, 611–624 10.1093/brain/awp306 20008375

[B74] Lombardo M. V. Chakrabarti B. Lai M.-C. MRC AIMS Consortium Baron-Cohen S. (2012). Self-referential and social cognition in a case of autism and agenesis of the corpus callosum. Mol. Autism 3, 14 10.1186/2040-2392-3-14 23171505 PMC3522057

[B75] Lynall M. E. Bassett D. S. Kerwin R. McKenna P. J. Kitzbichler M. Muller U. (2010). Functional connectivity and brain networks in schizophrenia. J. Neurosci. 30, 9477–9487 10.1523/JNEUROSCI.0333-10.2010 20631176 PMC2914251

[B76] Lynch C. J. Uddin L. Q. Supekar K. Khouzam A. Phillips J. Menon V. (2013). Default mode network in childhood autism: posteromedial cortex heterogeneity and relationship with social deficits. Biol. Psychiatry. 74, 212–219 10.1016/j.biopsych.2012.12.013 23375976 PMC3710546

[B77] Lyons V. Fitzgerald M. (2013). “Atypical sense of self in autism spectrum disorders: a neuro-cognitive perspective,” in Recent Advances in Autism Spectrum Disorders, Vol. 1, 749–770 10.5772/53680

[B78] Lysaker P. H. Lysaker J. T. (2010). Schizophrenia and alterations in self-experience: a comparison of 6 perspectives. Schizophr. Bull. 36, 331–340 10.1093/schbul/sbn077 18635676 PMC2833111

[B79] Macuga K. L. Frey S. H. (2011). Selective responses in right inferior frontal and supramarginal gyri differentiate between observed movements of oneself vs. another. Neuropsychologia 49, 1202–1207 10.1016/j.neuropsychologia.2011.01.005 21237185 PMC3078186

[B80] Marenco S. Stein J. L. Savostyanova A. A. Sambataro F. Tan H.-Y. Goldman A. L. (2013). Investigation of anatomical thalamo-cortical connectivity and fMRI activation in schizophrenia. Neuropsychopharmacology 37, 499–507 10.1038/npp.2011.215 21956440 PMC3242311

[B81] Matthews N. Gold B. J. Sekuler R. Park S. (2013). Gesture imitation in schizophrenia. Schizophr. Bull. 39, 94–101 10.1093/schbul/sbr062 21765171 PMC3523902

[B82] Mier D. Lis S. Esslinger C. Sauer C. Hagenhoff M. Ulferts J. (2013). Neuronal correlates of social cognition in borderline personality disorder. Soc. Cogn. Affect. Neurosci. 8, 531–537 10.1093/scan/nss028 22362841 PMC3682436

[B83] Modinos G. Ormel J. Aleman A. (2009). Activation of anterior insula during self-reflection. PLoS ONE 4:e4618 10.1371/journal.pone.0004618 19242539 PMC2643476

[B84] Monk C. S. Peltier S. J. Wiggins J. L. Weng S. J. Carrasco M. Risi S. (2009). Abnormalities of intrinsic functional connectivity in autism spectrum disorder. Neuroimage 47, 764–772 10.1016/j.neuroimage.2009.04.069 19409498 PMC2731579

[B85] Moran L. V. Tagamets M. A. Sampath H. O’Donnell A. Stein E. A. Kochunov P. (2013). Disruption of anterior insula modulation of large-scale brain networks in schizophrenia. Biol. Psychiatry. 10.1016/j.biopsych PMC3735654 23623456

[B86] Müller R. A. Shih P. Keehn B. Deyoe J. R. Leyden K. M. Shukla D. K. (2011). Underconnected, but how? A survey of functional connectivity MRI studies in autism spectrum disorders. Cereb. Cortex 21, 2233–2243 10.1093/cercor/bhq296 21378114 PMC3169656

[B87] New A. S. Hazlett E. A. Newmark R. E. Zhang J. Triebwasser J. Meyerson D. (2009). Laboratory induced aggression: a positron emission tomography study of aggressive individuals with borderline personality disorder. Biol. Psychiatry 66, 1107–1114 10.1016/j.biopsych.2009.07.015 19748078 PMC2788117

[B88] Ng S. H. Han S. Mao L. Lai J. C. L. (2010). Dynamic bicultural brains: fMRI study of their flexible neural representation of self and significant others in response to culture primes. Asian J. Soc. Psychol. 13, 83–91 10.1111/j.1467-839X.2010.01303.x

[B89] Nolen-Hoeksema S. Wisco B. E. Lyubomirsky S. (2008). Re-thinking rumination. Perspect. Psychol. Sci. 3, 400–424 10.1111/j.1745-6924.2008.00088.x 26158958

[B90] Northoff G. (2007). Psychopathology and pathophysiology of the self in depression – neuropsychiatric hypothesis. J. Affect. Disord. 104, 1–14 10.1016/j.jad.2007.02.012 17379318

[B91] Northoff G. Heinzel A. de Grech M. Bermpohl F. Dobrowolny H. Panksepp J. (2006). Self-referential processing in our brain – a meta-analysis of imaging studies on the self. Neuroimage 31, 440–457 10.1016/j.neuroimage.2005.12.002 16466680

[B92] Paul L. K. Brown W. S. Adolphs R. Tyszka J. M. Richards L. J. Mukherjee P. (2007). Agenesis of the corpus callosum: genetic, developmental and functional aspects of connectivity. Nat. Rev. Neurosci. 8, 287–299 10.1038/nrn2107 17375041

[B93] Philippi C. L. Feinstein J. S. Khalsa S. S. Damasio A. Tranel D. Landini G. (2012). Preserved self-awareness following extensive bilateral brain damage to the insula, anterior cingulate, and medial prefrontal cortices. PLoS ONE 7:e38413 10.1371/journal.pone.0038413 22927899 PMC3425501

[B94] Pizzagalli D. A. (2011). Frontocingulate dysfunction in depression: toward biomarkers of treatment response. Neuropsychopharmacology 36, 183–206 10.1038/npp.2010.166 20861828 PMC3036952

[B95] Posey D. J. Kem D. L. Swiezy N. B. Sweeten T. L. Wiegand R. E. McDougle C. J. (2004). A pilot study of D-cycloserine in subjects with autistic disorder. Am. J. Psychiatry 161, 2115–2117 10.1176/appi.ajp.161.11.2115 15514414

[B96] Qin P. Northoff G. (2011). How is our self related to midline regions and the default-mode network? Neuroimage 57, 1221–1233 10.1016/j.neuroimage.2011.05.028 21609772

[B97] Raballo A. Saebye D. Parnas J. (2011). Looking at the schizophrenia spectrum through the prism of self-disorders: an empirical study. Schizophr. Bull. 37, 344–351 10.1093/schbul/sbp056 19528205 PMC3044618

[B98] Rizzolatti G. Fabbri-Destro M. (2010). Mirror neurons: from discovery to autism. Exp. Brain Res. 200, 223–237 10.1007/s00221-009-2002-3 19760408

[B99] Rotarska-Jagiela A. van de Ven V. Oertel-Knöchel V. Uhlhaas P. J. Vogeley K. Linden D. E. J. (2010). Resting-state functional network correlates of psychotic symptoms in schizophrenia. Schizophr. Res. 117, 21–30 10.1016/j.schres.2010.01.001 20097544

[B100] Ruocco A. C. Amirthavasagam S. Choi-Kain L. W. McMain S. F. (2012). Neural correlates of negative emotionality in borderline personality disorder: an activation-likelihood-estimation meta-analysis. Biol. Psychiatry 73, 153–160 10.1016/j.biopsych.2012.07.014 22906520

[B101] Ruocco A. C. Medaglia J. D. Tinker J. R. Ayaz H. Forman E. M. Newman C. F. (2010). Medial prefrontal cortex hyperactivation during social exclusion in borderline personality disorder. Psychiatry Res. 181, 233–236 10.1016/j.pscychresns.2009.12.001 20153143

[B102] Rüsch N. Bracht T. Kreher B. W. Schnell S. Glauche V. Il’yasov A. (2010). Reduced interhemispheric structural connectivity between anterior cingulate cortices in borderline personality disorder. Psychiatry Res. 181, 151–154 10.1016/j.pscychresns.2009.08.004 20079614

[B103] Rüsch N. Luders E. Lieb K. Zahn R. Ebert D. Thompson P. M. (2007). Corpus callosum abnormalities in women with borderline personality disorder and co-morbid attention-deficit hyperactivity disorder. J. Psychiatry Neurosci. 32, 417–422 18043765 PMC2077349

[B104] Salanova V. Andermann F. Rasmussen T. Olivier A. Quesney L. F. (1995). Parietal lobe epilepsy. Clinical manifestations and outcome in 82 patients treated surgically between 1929 and 1988. Brain 118, 607–627 10.1093/brain/118.3.607 7600082

[B105] Salavert J. Gasol M. Vieta E. Cervantes A. Trampal C. Gispert J. D. (2011). Fronto-limbic dysfunction in borderline personality disorder: a 18F-FDG positron emission tomography study. J. Affect. Disord. 131, 260–267 10.1016/j.jad.2011.01.001 21272937

[B106] Sass L. A. Parnas J. (2003). Schizophrenia, consciousness, and the self. Schizophr. Bull. 29, 427–444 10.1093/oxfordjournals.schbul.a007017 14609238

[B107] Scheidegger M. Walter M. Lehmann M. Metzger C. Grimm S. Boeker H. (2012). Ketamine decreases resting state functional network connectivity in healthy subjects: implications for antidepressant drug action. PLoS ONE 7:e44799 10.1371/journal.pone.0044799 23049758 PMC3461985

[B108] Schneider K. (1959). Clinical Psychopathology, trans. Hamilton M. W. New York, NY: Grune and Stratton

[B109] Schulte-Rüther M. Greimel E. Piefke M. Kamp-Becker I. Remschmidt H. Fink G. R. (2013). Age dependent changes in the neural substrates of empathy in Autism spectrum disorder. Soc. Cogn. Affect. Neurosci. 10.1093/scan/nst088 23784073 PMC4127013

[B110] Schwartz G. E. Fair P. L. Salt B. A. Mandel M. R. Klerman G. L. (1976). Facial expression and imagery in depression: an electromyographic study. Psychosom. Med. 38, 337–347 981492 10.1097/00006842-197609000-00006

[B111] Shad M. U. Keshavan M. S. Steinberg J. L. Mihalakos P. Thomas B. P. Motes M. A. (2012). Neurobiology of self-awareness in schizophrenia: an fMRI study. Schizophr. Res. 138, 113–119 10.1016/j.schres.2012.03.016 22480958 PMC3372627

[B112] Sheline Y. I. Barch D. M. Price J. L. Rundle M. M. Vaishnavi S. N. Synder A. Z. (2009). The default mode and self-referential processes in depression. Proc. Natl. Acad. Sci. U.S.A. 106, 1942–1947 10.1073/pnas.0812686106 19171889 PMC2631078

[B113] Sheline Y. I. Price J. L. Yan Z. Mintun M. A. (2010). Resting-state functional MRI in depression unmasks increased connectivity between networks via the dorsal nexus. Proc. Natl. Acad. Sci. U.S.A. 107, 11020–11025 10.1073/pnas.1000446107 20534464 PMC2890754

[B114] Silani G. Bird G. Brindley R. Singer T. Frith C. Frith U. (2008). Levels of emotional awareness and autism: an fMRI study. Soc. Neurosci. 3, 97–112 10.1080/17470910701577020 18633852

[B115] Silk T. J. Rinehart N. Bradshaw J. L. Tonge B. Egan G. O’Boyle M. W. (2006). Visuospatial processing and the function of prefrontal-parietal networks in autism spectrum disorders: a functional MRI study. Am. J. Psychiatry 163, 1440–1443 10.1176/appi.ajp.163.8.1440 16877661

[B116] Sliz D. Hayley S. (2012). Major depressive disorder and alterations in insular cortical activity: a review of current functional magnetic imaging research. Front. Hum. Neurosci. 6:323 10.3389/fnhum.2012.00323 23227005 PMC3512092

[B117] Sommer I. E. C. Diederen K. M. J. Blom J.-D. Willems A. Kushan L. Slotema K. (2008). Auditory verbal hallucinations predominantly activate the right inferior frontal area. Brain 131, 3169–3177 10.1093/brain/awn251 18854323

[B118] Stephan K. E. Friston K. J. Frith C. D. (2009). Dysconnection in schizophrenia: from abnormal synaptic plasticity to failures of self-monitoring. Schizophr. Bull. 35, 509–527 10.1093/schbul/sbn176 19155345 PMC2669579

[B119] Stewart J. L. Coan J. A. Towers D. N. Allen J. J. B. (2011). Resting frontal EEG asymmetry during emotional challenge differentiates individuals with and without lifetime major depressive disorder. J. Affect. Disord. 129, 167–174 10.1016/j.jad.2010.08.029 20870293 PMC3021630

[B120] Synofzik M. Thier P. Leube D. T. Schlotterbeck P. Lindner A. (2010). Misattributions of agency in schizophrenia are based on imprecise predictions about the sensory consequences of one’s actions. Brain 133, 262–271 10.1093/brain/awp291 19995870

[B121] Tao H. Guo S. Ge T. Kendrick K. M. Xue Z. Liu Z. (2011). Depression uncouples brain hate circuit. Mol. Psychiatry. 18, 101–111 10.1038/mp.2011.127 21968929 PMC3526729

[B122] Torrey E. F. (2007). Schizophrenia and the inferior parietal lobule. Schizophr. Res. 97, 215–225 10.1016/j.schres.2007.08.023 17851044

[B123] Treadway M. T. Zald D. H. (2011). Reconsidering anhedonia in depression: lessons from translational neuroscience. Neurosci. Biobehav. Rev. 35, 537–555 10.1016/j.neubiorev.2010.06.006 20603146 PMC3005986

[B124] Tsai G. E. Lin P.-Y. (2010). Strategies to enhance N-methyl-D-aspartate receptor-mediated neurotransmission in schizophrenia, a critical review and meta-analysis. Curr. Pharm. Des. 16, 522–537 10.2174/138161210790361452 19909229

[B125] Uddin L. Q. (2011). Brain connectivity and the self: the case of cerebral disconnection. Conscious. Cogn. 20, 94–98 10.1016/j.concog.2010.09.009 20875750 PMC3021584

[B126] Uddin L. Q. Iacobini M. Lange C. Keenan J. P. (2007). The self and social cognition: the role of cortical midline structures and mirror neurons. Trends Cogn. Sci. (Regul. Ed.) 11, 153–157 10.1016/j.tics.2007.01.001 17300981

[B127] Uddin L. Q. Menon V. (2009). The anterior insula in autism: under-connected and under-examined. Neurosci. Biobehav. Rev. 33, 1198–1203 10.1016/j.neubiorev.2009.06.002 19538989 PMC2743776

[B128] Uddin L. Q. Molnar-Szakacs I. Zaidel E. Iacobini M. (2006). rTMS to the right inferior parietal lobule disrupts self-other discrimination. Soc. Cogn. Affect. Neurosci. 1, 65–71 10.1093/scan/nsl003 17387382 PMC1832105

[B129] van der Meer L. de Vos A. E. Stiekema A. P. M. Pijnenborg G. H. M. van Tol M.-J. Nolen W. A. (2012). Insight in schizophrenia: involvement of self-reflection networks? Schizophr. Bull. 10.1093/schbul/sbs122 23104865 PMC3796073

[B130] Vanhaudenhuyse A. Noirhomme Q. Tshibanda L. J.-F. Bruno M.-A. Boveroux P. Schnakers C. (2010). Default network connectivity reflects the level of consciousness in non-communicative brain-damaged patients. Brain 133, 161–171 10.1093/brain/awp313 20034928 PMC2801329

[B131] Vissers M. E. Cohen M. X. Geurts H. M. (2012). Brain connectivity and high functioning autism: a promising path of research that needs refined models, methodological convergence, and stronger behavioral links. Neurosci. Biobehav. Rev. 36, 604–625 10.1016/j.neubiorev.2011.09.003 21963441

[B132] Voss M. Moore J. Hauser M. Gallinat J. Heinz A. Haggard P. (2010). Altered awareness of action in schizophrenia: a specific deficit in predicting action consequences. Brain 133, 3104–3112 10.1093/brain/awq152 20685805

[B133] Walterfang M. Chanen A. M. Barton S. Wood A. G. Jones S. Reutens D. C. (2010). Corpus callosum morphology and relationship to orbitofrontal and lateral ventricular volume in teenagers with first presentation borderline personality disorder. Psychiatry Res. 183, 30–37 10.1016/j.pscychresns.2010.04.001 20605421

[B134] Wang L. Hermens D. F. Hickie I. B. Lagopoulos J. (2012). A systematic review of resting-state functional-MRI studies in major depression. J. Affect. Disord. 142, 6–12 10.1016/j.jad.2012.04.013 22858266

[B135] Waters F. Woodward T. Allen P. Aleman A. Sommer I. (2012). Self-recognition deficits in schizophrenia patients with auditory hallucinations: a meta-analysis of the literature. Schizophr. Bull. 38, 741–750 10.1093/schbul/sbq144 21147895 PMC3406529

[B136] Waters F. A. V. Badcock J. C. (2010). First-rank symptoms in schizophrenia: reexamining mechanisms of self-recognition. Schizophr. Bull. 26, 510–517 10.1093/schbul/sbn112 18753307 PMC2879682

[B137] Welsh R. C. Chen A. C. Taylor S. F. (2010). Low-frequency BOLD fluctuations demonstrate altered thalamocortical connectivity in schizophrenia. Schizophr. Bull. 36, 713–722 10.1093/schbul/sbn145 18990709 PMC2894601

[B138] Weng S.-J. Wiggins J. L. Peltier S. J. Carrasco M. Risi S. Lord C. (2010). Alterations of resting state functional connectivity in the default network in adolescents with autism spectrum disorders. Brain Res. 1313, 202–214 10.1016/j.brainres.2009.11.057 20004180 PMC2818723

[B139] Whitford T. J. Kubicki M. Schneiderman J. S. O’Donnell L. J. King R. Alvarado J. L. (2010). Corpus callosum abnormalities and their associations with psychotic symptoms in patients with schizophrenia. Biol. Psychiatry 68, 70–77 10.1016/j.biopsych.2010.03.025 20494336 PMC2900500

[B140] Wiebking C. Bauer A. de Greck M. Duncan N. W. Tempelmann C. Northoff G. (2010). Abnormal body perception and neural activity in the insula in depression: an fMRI study of the depressed “material me”. World J. Biol. Psychiatry 11, 538–549 10.3109/15622970903563794 20146653

[B141] Wiggins J. L. Peltier S. J. Ashinoff S. Weng S.-J. Carrasco M. Welsh R. C. (2011). Using a self-organizing map algorithm to detect age-related changes in functional connectivity during rest in Autism spectrum disorders. Brain Res. 1380, 187–197 10.1016/j.brainres.2010.10.102 21047495 PMC3050117

[B142] Wilkinson-Ryan T. Westen D. (2000). Identity disturbance in borderline personality disorder: an empirical investigation. Am. J. Psychiatry 157, 528–541 10.1176/appi.ajp.157.4.528 10739411

[B143] Wolf R. C. Sambataro F. Vasic N. Schmid M. Thomann P. A. Bienentreu S. D. (2011). Aberrant connectivity of resting-state networks in borderline personality disorder. J. Psychiatry Neurosci. 36, 402–411 10.1503/jpn.100150 21406160 PMC3201994

[B144] Xu K. Jiang W. Ren L. Ouyang X. Jiang Y. Wu F. (2013). Impaired interhemispheric connectivity in medication-naive patients with major depressive disorder. J. Psychiatry Neurosci. 38, 43–48 10.1503/jpn.110132 22498077 PMC3529218

[B145] Zhou Y. Liang M. Jiang T. Tian L. Liu Y. Liu Z. (2007). Functional dysconnectivity of the dorsolateral prefrontal cortex in first-episode schizophrenia using resting-state fMRI. Neurosci. Lett. 417, 297–302 10.1016/j.neulet.2007.02.081 17399900

[B146] Zhu X. Wang X. Xiao J. Liao J. Zhong M. Wang W. (2012). Evidence of a dissociation in resting-state default mode network connectivity in first-episode, treatment naive major depression patients. Biol. Psychiatry 71, 611–617 10.1016/j.biopsych.2011.10.035 22177602

[B147] Zhu Y. Zhang L. Fan J. Han S. (2007). Neural basis of cultural influence on self representation. Neuroimage 34, 1310–1317 10.1016/j.neuroimage.2006.08.047 17134915

